# Comparison of Maillard-Type Glycated Collagen with Alginate Oligosaccharide and Glucose: Its Characterization, Antioxidant Activity, and Cytoprotective Activity on H_2_O_2_-Induced Cell Oxidative Damage

**DOI:** 10.3390/foods11152374

**Published:** 2022-08-08

**Authors:** Boxue Yang, Ga-Hyun Joe, Wenzhao Li, Yutaka Shimizu, Hiroki Saeki

**Affiliations:** Faculty of Fisheries Sciences, Hokkaido University, Minato 3, Hokkaido, Hakodate 041-8611, Japan

**Keywords:** collagen, Maillard reaction, alginate oligosaccharide, antioxidant activity, oxidative stress protection

## Abstract

To improve the antioxidant activity of collagen molecules using Maillard-type glycation, the relation between antioxidant activity and progress indexes for the Maillard reaction must be understood. In this study, lyophilized tilapia scale collagen was mixed with a half weight of alginate oligosaccharide (AO) or glucose and incubated at 60 °C and 35% relative humidity for up to 18 h to produce the Maillard-type glycated collagen (C-AO and C-Glu, respectively). As glycation progressed, the amount of conjugated sugar coupled with UV-vis absorbance at 294 nm and 420 nm increased more rapidly in C-Glu than in C-AO, and the available lysine decreased rapidly in C-Glu compared with C-AO. The early-to-middle- and late-stage products of the Maillard reaction were involved in enhanced antioxidant activity of digested C-AO and digested C-Glu, respectively. Additionally, C-AO acquired the antioxidant activity without marked available lysine loss. The cytoprotective effect of collagen in H_2_O_2_-induced damage was enhanced by glycation, achieved by reducing malondialdehyde content and increasing superoxide dismutase and catalase activities. These results indicate that AO is an excellent reducing sugar that enhances the health benefits of collagen without excessive loss of lysine, which is a nutritional problem of the Maillard-type glycation.

## 1. Introduction

Previous studies have illustrated that excess free radicals in the body are associated with various serious diseases, such as cardiovascular diseases and cancer [1]. Therefore, eliminating free radicals from the body via the intake of antioxidants can reduce oxidative stress and suppress the exacerbation of associated diseases [1,2]. It has been reported that the antioxidant components present in various foods have beneficial effects on human health, and several of these antioxidants have been identified [1,2]. Additionally, protein glycation using the Maillard reaction effectively improved the functions of the food proteins, especially their antioxidant activity [3,4]. Thus far, numerous reducing sugars, including monosaccharides [5], oligosaccharides [6], and polysaccharides [7], have been employed in Maillard-type glycation to enhance the antioxidant activities of various food proteins, such as casein [5], whey protein [6], and longan pulp protein [7].

Alginate oligosaccharide (AO) is derived from alginate, and it contains the major polysaccharides of brown algae, including β-d-mannuronate and α-l-guluronate, at different degrees of polymerization [8]. Because AO is a multifunctional oligosaccharide, it has been extensively employed in the food industry, medicine, and agriculture [9]. Some studies have reported improvements in the following food functions after the application of AO-based Maillard-type glycation (abbreviated as AO-glycation): solubility [10,11,12,13,14], thermal stability [10,13,14,15], emulsion-forming ability [13,15], and anti-inflammatory activity [16,17,18]. However, there are no examples regarding the improving effect of AO-glycated protein on antioxidant activity, which is a remission factor for various non-communicable diseases [1,2].

In global fishery production, aquaculture has been playing an increasing role in the supply of aquatic proteins [19]. Tilapia is a typical fish species in land-based aquaculture. Its global aquaculture production was approximately 5.71 million tons in 2017, which represented 10.14% of the total cultured fish production [20]. Moreover, the scale, bone, and skin of the fish are byproducts of fish processing, representing 30% of the total fish weight [21]. Collagen, the subject protein of this research, is an important material in the food industry [22], with the byproducts of fishery possessing high levels of collagen [23]. The enhanced antioxidant activity induced by Maillard-type glycation was observed in collagen peptides and gelatin hydrolysates glycated with monosaccharides [24,25]. In addition, in the amino acid–sugar reaction model [26,27] and the porcine plasma protein–glucose conjugate [28], antioxidant activity was enhanced with the progression of the Maillard reaction. However, no clear correlation was observed between antioxidant activity and the Maillard reaction indicator in the casein–ribose reaction system [5], suggesting this correlation varied with the protein or sugar type used in the reaction. Furthermore, the correlation between enhanced antioxidant activity and Maillard reaction indicators in glycated collagen has not been fully explained. Therefore, to appropriately control collagen-based Maillard-type glycation and expand the application of the antioxidant glycated collagen, the relationship between various Maillard reaction indicators and the acquired antioxidant activity should be investigated in detail.

The objectives of this study were to investigate the effect of Maillard-type glycation with AO or glucose on the antioxidant activity of collagen; the following points were examined in the present study: (1) characterization of AO and glucose glycated collagens; (2) comparison of the antioxidant activity between AO and glucose glycated collagen, which referred to various Maillard reaction indicators at different reaction time; (3) investigation of the cytoprotective activity on H_2_O_2_-induced cellular oxidative stress, which involved the production of malondialdehyde (MDA) and the activity of superoxide dismutase (SOD) and catalase (CAT).

## 2. Materials and Methods

### 2.1. Materials

Tilapia scale collagen (acid soluble atelocollagen ATH-221, purity > 99.99%, purchased from Koken Co., Ltd., Tokyo, Japan) and AO (mean degree of polymerization = 6, mean molecular mass: 1.1 kDa, prepared by alginate lyase degradation, and supplied from Hokkaido Mitsui Chemical Industry Co., Ltd., Hokkaido, Japan) were used in this study. Pepsin from porcine gastric mucosa and trypsin from a bovine pancreas was then purchased from Sigma-Aldrich (St. Louis, MO, USA), whereas 1,1-diphenyl- 2-picrylhydrazyl (DPPH), and 2,2′-azino-bis(3-ethylbenzothiazoline-6-sulfonic acid) diammonium salt (ABTS) were purchased from Tokyo Chemical Industry Co., (Tokyo, Japan) and Fujifilm Wako Pure Chemical Co., (Tokyo, Japan), respectively. In addition, RAW264.7 cells (murine macrophage cell line) were purchased from the American Type Culture Collection via Dainippon Sumitomo Pharma Co., Ltd. (Osaka, Japan). Thirty percent hydrogen peroxide solution was purchased from Fujifilm Wako Pure Chemical Co., (Tokyo, Japan). Moreover, all other chemicals with no descriptions in the manuscript were purchased from Kanto Chemical Co., Inc. (Tokyo, Japan), Kagaku, or Wako Pure Chemical Industries Ltd. (Osaka, Japan).

### 2.2. Glycation of Collagen

The collagen–sugar conjugates, C-AO (glycated with AO) and C-Glu (glycated with glucose), were prepared in the present study, and a collagen–sorbitol mixture (C-Sor) was also incubated under similar conditions as a control. The collagen was glycated with reducing sugars, as described by Nishizawa et al., with slight modifications [16]. First, the collagen was suspended in distilled water at a concentration of 2 mg/mL and mixed with sorbitol (C-Sor), AO and sorbitol (C-AO), or glucose (C-Glu), and the weight ratio of collagen to each sugar was set to 1:0.5. In this study, similar to the cryoprotective effect of glucose, sorbitol was employed as a non-reducing sugar to protect the collagen during the freezing stage. Subsequently, the collagen–sugar mixture was homogenized using a Potter-Elvehjem homogenizer and lyophilized using a freeze-dryer (FDU-2200, Tokyo Rikakikai, Co., Inc., Tokyo, Japan) until the following step. Afterward, the lyophilized collagen–sugar mixture was incubated at 60 °C with 35% relative humidity for 0 h, 6 h, 12 h, and 18 h, respectively, using a temperature–humidity cabinet (Sh-220, Tabai ESPEC Corp., Tokyo, Japan) to attach AO and glucose to collagen through the Maillard reaction.

After removing unreacted sugars using an ethanol treatment, these glycated collagens (C-AO and C-Glu, C-Sor as control) were used in the subsequent experiments. Briefly, glycated collagens containing unreacted sugars were suspended at 1 mg/mL in distilled water with a homogenizer (Ultra-Turrax T25, IKA, Staufen, Germany). After the addition of 80% ethanol at the final concentration, each homogenate was cooled at −20 °C for 1 h and centrifuged at 20,000× *g* for 30 min. Finally, the precipitated collagen was collected and lyophilized as washed glycated collagen and then stored at −60 °C until use.

### 2.3. Measurement of UV-Vis Absorbance and Browning Intensity

The lycated collagens were dissolved in 10 mM of HCl (pH 2.0) at 2 mg/mL using ultrasonication. While the UV-vis absorbance at 294 nm (A294) was used as an indicator of the intermediate Maillard reaction stage, that at 420 nm (A420) was used as an indicator of browning intensity [29]. The measurements were conducted using a UV-Visible spectrophotometer (V-630, Jasco, Japan).

### 2.4. Measurement of Available Lysine Content

The glycated collagens were suspended in 50 mM of sodium phosphate buffer (pH 9.5) containing 2% sodium dodecyl sulfate (SDS). Afterward, ultrasonication was performed for the complete dissolution of the collagen. Subsequently, the mixture was subjected to the available lysine assay using *o*-phthalaldehyde and N-acetyl-K-cysteine [30]. The protein concentration was measured by the Lowry method [31], and the available lysine content was expressed as a relative value with non-incubated C-Sor.

### 2.5. Measurement of Sugars Bound to Collagen

The number of total sugars bound to collagen was measured using the phenol–sulfuric acid method as described by Nishizawa et al. with slight modification [16,32]. For C-AO and C-Glu measurements, the standard calibration curve was prepared using AO and glucose, respectively. The protein concentration was measured using the Lowry method as described in Section 2.4. To compare the amount of sugar in each glycated collagen sample, the results were expressed as nmol/mg (sugar/protein).

### 2.6. SDS-Polyacrylamide Gel Electrophoresis (SDS-PAGE)

The glycated collagen was suspended in distilled water, mixed with an equal volume of SDS-regent (2% SDS, containing 8 M urea, and 2% β-mercaptoethanol and 20 mM Tris–HCl (pH 8.0)) at a final concentration of 1 mg/mL, then heated with boiled water for 2 min. Subsequently, each 10 µL sample was subjected to SDS-PAGE using 7.5% polyacrylamide slab-gel, according to the method of Leammli [33]. Then, the electrophoresed gel was stained using Coomassie Brilliant Blue R-250 (Bio-Rad Laboratories, Hercules, CA, USA).

### 2.7. Simulated Gastrointestinal Digestion In Vitro

The glycated collagen was continuously digested using pepsin and trypsin [16] to estimate the antioxidant and cytoprotective activities induced by oxidative damage. Briefly, several glycated collagens and C-Sor at 2 mg/mL were digested using pepsin (pH 2.0) and trypsin (pH 8.0), each for 3 h at 37 °C. The proteases were added to the collagens at 1/100 of the protein content, after which the pH was adjusted with 1 M HCl and 1 M NaOH. Afterward, the digested collagens were boiled for 15 min to terminate the digestion, followed by centrifugation at 20,000× *g* for 30 min. The digested collagens were collected in the supernatant, lyophilized, and stored at −20 °C until they were subjected to the antioxidant activity and cytoprotective assay described below.

### 2.8. Analysis of Molecular Mass Distribution

After the in vitro-simulated gastrointestinal digestion and lyophilization, the digested collagens were subjected to molecular mass distribution analysis. Analysis was performed using a gel permeation chromatography coupled with a Superdex Peptide 10/300 GL column (GE Healthcare Life Science, Little Chalfont, UK). First, the digested collagens were dissolved in 50 mM NaCl at a final concentration of 5 mg/mL and filtered with a 0.45–μm membrane. The chromatograph operation conditions were as follows: 50 mM NaCl as the eluent at a 0.5 mL/min flow rate and detection wavelength of 220 nm. Finally, the molecular mass distribution of digested peptides was expressed as a percentage of the total peak area by classifying the obtained chromatograms into four types of molecular mass ranges (>10 kDa, 10–5 kDa, 5–1 kDa, and <1 kDa). Parvalbumin (12 kDa), aprotinin (6.512 kDa), cyanocobalamin (1.355 kDa), and hippuric acid (179 Da) were used as molecular mass markers.

### 2.9. Measurement of Antioxidant Activity

#### 2.9.1. DPPH Radical Scavenging Assay

A DPPH assay of digested collagens was conducted as described by Li et al. [34] with minor modifications. First, the digested collagens were dissolved in distilled water at 10 mg/mL, mixed with an equal volume of 0.115 mM DPPH in methanol. Subsequently, the absorbance at 517 nm was measured after keeping the samples in the dark at room temperature for 30 min (*A_sample_*). DPPH radical scavenging activity was calculated using the following equation:

$$DPPH\;radical\;scavenging\;activity\;\left(\%\right)=\left(1-\frac{A_{sample}-A_{background}}{A_{control}}\right)\times 100$$

where, *A_background_* and *A_control_* were the absorbances of the digested collagens dissolved in distilled water at 5 mg/mL and the 115 µM of DPPH in methanol diluted with equal volumes of distilled water, respectively.

#### 2.9.2. ABTS Radical Scavenging Assay

An ABTS assay of the digested collagens was performed as described by Li et al. [34] with a slight modification. First, cation-radical ABTS was generated by mixing 7 mM of ABTS and 2.45 mM of potassium persulfate at a mixing ratio of 2:1 (*v*/*v*). The mixture was incubated at room temperature in the dark for 12–16 h and diluted with 10 mM of phosphate-buffered saline (PBS, pH 7.4) to adjust the absorbance to 0.70 ± 0.02 at 734 nm (referred to as ABTS working solution). Afterward, the digested collagens dissolved in PBS at 0.5 mg/mL reacted with an equal volume of the ABTS working solution at room temperature in the dark for 10 min. Finally, absorbance was measured at 734 nm (*A_sample_*), after which the ABTS radical scavenging activity was calculated using the following equation:

$$ABTS\;radical\;scavenging\;activity\;\left(\%\right)=\left(1-\frac{A_{sample}-A_{background}}{A_{control}}\right)\times 100$$

where, *A_background_* and *A_control_* were the absorbances of the digested collagens dissolved in PBS at 0.25 mg/mL and that of ABTS working solution diluted double with PBS, respectively.

### 2.10. Cell Culture

The raw 264.7 cells were cultured in Dulbecco’s modified Eagle’s medium (DMEM), containing 10% heat-inactivated fetal bovine serum (FBS), 100 units/mL of penicillin, 100 μg/mL of streptomycin, and 0.1 mM of non-essential amino acids at 37 °C in a 5% CO_2_ humidified atmosphere.

### 2.11. Measurement of Cell Viability

The RAW 264.7 cells were seeded in a 96-well plate at 2 × 10^4^ cells/well and incubated with DMEM containing 10% FBS for 2 h at 37 °C in 5% CO_2_. Subsequently, the cells were cultured with DMEM containing 0.1% FBS for 24 h for serum starvation. After performing serum starvation, these cells were treated with 200 μg/mL of the digested C-AO or C-Glu (0-h or 18-h glycation) in DMEM containing 0.1% FBS for 24 h, after which the cytotoxicity of the glycated collagen digest was measured using a cell viability/cytotoxicity assay kit (CCK-8, Dojindo Laboratories, Kumamoto, Japan) according to the manufacturer’s protocol.

### 2.12. Cytoprotective Effect of Glycated Collagens on H_2_O_2_-Induced Oxidative Damage

The cells were seeded at 2 × 10^4^ cells/well on a 96-well plate and cultured in DMEM containing 10% FBS for 2 h. After performing serum starvation, as described in Section 2.11, DMEM containing 0.1% FBS was removed. The cells were treated with 50 or 200 μg/mL of the digested C-AO or C-Glu (0-h or 18-h glycation) in DMEM with 0.1% FBS for 24 h. After removing the digested C-AO or C-Glu by medium exchange, the cells were cultured in the same medium containing 0.8 mM H_2_O_2_ for 4 h. Afterward, the cells were washed with PBS and DMEM containing 0.1% FBS was re-added. Finally, the cell viability was measured by the method described in Section 2.11. The viability of the cells that were not pretreated with glycated collagen and 0.8 mM H_2_O_2_ was set to 100% to evaluate the cytoprotective effect of glycated collagen. Prior to the experiment, the cells of the same density were cultured in DMEM containing 0.1% FBS with 0.2–1.0 mM H_2_O_2_ for 4 h to identify the optimum H_2_O_2_ concentration for the assessment [35].

### 2.13. Measurement of SOD, CAT, and MDA in H_2_O_2_-Induced Raw 264.7 Cells

The activity of superoxide dismutase (SOD), catalase (CAT), and the content of malondialdehyde (MDA) were measured using an SOD Assay Kit-WST (#S311, Dojindo Laboratories, Kumamoto, Japan), Catalase Colorimetric Activity Kit (#K033-H1, Arbor Assay, Ann Arbor, MI, USA), and MDA Assay Kit (#M496, Dojindo Laboratories), respectively. The measurements were conducted according to the manufacturers’ instructions.

The cells were cultured in 6-cm dishes at a density of 2 × 10^6^ cells/dish, and the pretreatment and exposure to H_2_O_2_ to induce oxidative stress were performed, as described in Section 2.12. Then, the cells were collected and lysed following the manufacturer’s protocols. After centrifugation at 10,000× *g* for 30 min at 4 °C, the supernatant was used for each measurement. The protein concentration in each supernatant was determined using a Protein Assay Rapid Kit (Fujifilm Wako Pure Chemical Co., Tokyo, Japan).

SOD activity was defined as the amount of the enzyme in 20 µL of the sample solution that inhibited the reduction reaction of WST-1 with superoxide anion by 50%, as per the manufacturer’s instructions. After normalization to the protein concentration, the result was expressed as U/mg protein.

CAT activity and MDA content were calculated using the standard calibration curve supplied in the respective kits. The results for CAT and MDA were also normalized to the protein concentration and expressed as U/mg protein and nmol/mg protein, respectively.

### 2.14. Statistical Analysis

The data were subjected to the Tukey–Kramer test using JMP software (version 13, SAS Institute Inc., Cary, NC, USA). In addition, relations between antioxidant activity and various Maillard reaction indicators were performed using multivariate relations and linear regression analysis using JMP software. The data were expressed as mean ± standard deviation, and *p* < 0.05 was considered significant. All of the experiments were conducted thrice independently, and the cell experiments were performed independently five times.

## 3. Results and Discussion

### 3.1. The Introduction of Reducing Sugars to Collagen through the Maillard Reaction

**Figure 1:**
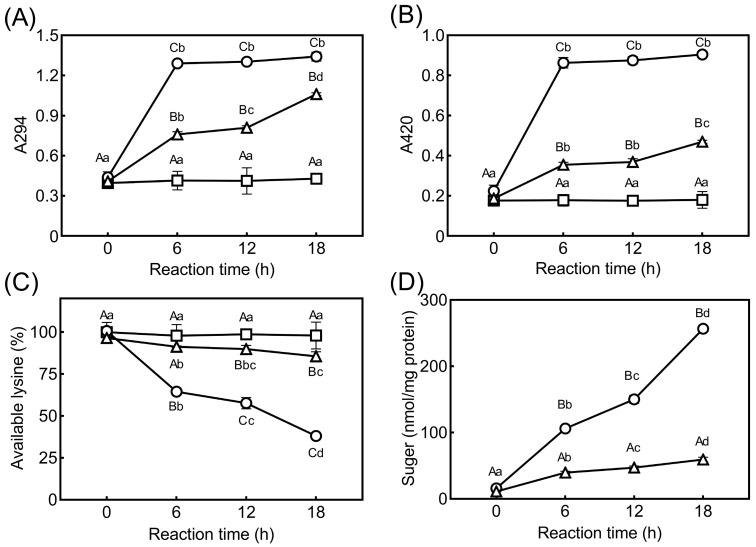
Progressing collagen glycation with AO and Glu monitored by indicators of the Maillard reaction. (**A**): UV-vis absorbance at 294 nm (A294); (**B**): UV-vis absorbance at 420 nm (A420); (**C**): available lysine content; (**D**): the amount of reducing sugars bound to collagen. C-Glu (○), C-AO (△), and C-Sor (□). Different capital letters A–C indicate significant differences among the three sugar groups at the same reaction time. Different lowercase letters a–d show significant differences in the effect of the reaction time within the same collagen–sugar group.

The progress of glycating collagen with AO and glucose was investigated by monitoring the indicators of the Maillard reaction, as shown in Figure 1A,B. The UV-vis absorbance at 294 nm (A294) and 420 nm (A420) in C-AO and C-Glu increased as the incubation time progressed. A marked available lysine loss was simultaneously observed in both groups (Figure 1C), whereas no marked change was observed in other amino acids except for arginine with a slight decrease (data not shown). In the concern of lysine and arginine loss in the collagen glycation, the reducing sugars bound to collage were measured and presented in Figure 1D, and it could be confirmed that the amount of conjugated reducing sugars increased with the progress of the glycation. Conversely, no absorbance increase and available lysine loss were observed in C-Sor, containing sorbitol, a non-reducing sugar. These results indicate that AO and glucose were conjugated with collagen via the Maillard reaction and the generated glycated collagen molecules as a result.

Although the available lysine loss of the C-AO group decreased gradually to 91% and 85% after incubation for 6 h and 18 h, respectively (Figure 1C), a marked decrease in the lysine loss occurred in C-Glu. As observed, the reduction rate of available lysine in C-Glu was 4.7 times higher than in C-AO after the 6-h reaction, which decreased to 36% after 18 h. In contrast, even though A294 and A420 in C-AO increased continuously even at the 18-h reaction, they increased rapidly and remained practically unchanged after 6 h in C-Glu (Figure 1A,B). The amount of AO and glucose binding to collagen continuously increased during the reaction; however, the amount of bound glucose was significantly higher than that of AO after 6 h of the reaction (Figure 1D). Therefore, the results in Figure 1 indicate that AO-based glycation progressed more slowly than glucose-based glycation.

### 3.2. The Molecular Mass Distribution of Collagen-Sugar Conjugates and Their Digested Peptides

**Figure 2:**
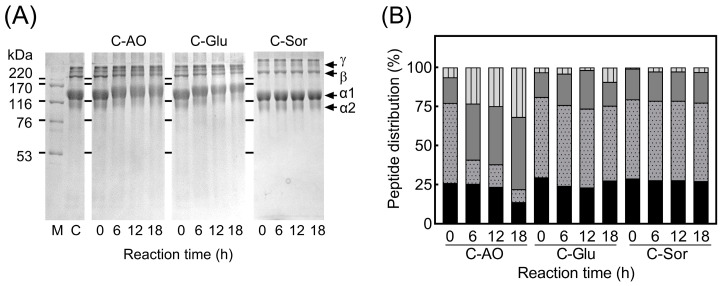
Molecular mass profiles of glycated collagens and their pepsin-trypsin digested peptides. (**A**): SDS-PAGE of glycated collagen. M: molecular mass markers; C: intact collagen. α1 and α2 indicate collagen subunits, β and γ are dimer and trimer chains of collagen subunits, respectively. (**B**): Gel permeation chromatography of digested glycosylated collagens. In the bar chart of molecular mass distribution, digested peptides were represented as follows: >10 kDa (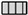), 10–5 kDa (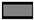), 5–1 kDa (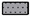), and <1 kDa (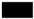).

The changes in the subunit component of collagen during glycation were examined using SDS-PAGE. As presented in Figure 2A (Lane C), the collagen on the tilapia scales comprised two α chains (α1 and α2) with a molecular mass of approximately 130 and 115 kDa, respectively. Additionally, the β (dimers) and γ (trimers) chains were present. These band patterns agreed with those of typical fish type I collagen [36].

No protein fragmentation was observed in C-AO, C-Glu, and C-Sor, indicating that the primary structure of collagen remained unchanged during the glycation process. However, the mobility and dyeing intensity of C-AO and C-Glu decreased with glycation, and this was not observed for C-Sor. These were the typical changes observed in the SDS-PAGE pattern of glycated proteins obtained using the Maillard reaction [12]. Hence, the decrease in the mobility of collagen subunits in C-AO and C-Glu is proposed to be caused by the increasing molecular mass of collagen, including the conjugation of AO or glucose with collagen during the dry-heating step in the Maillard reaction (Figure 1D) [37]. In addition, the results presented in Figure 2A and the increase in the browning intensity highlighted in Figure 1B suggest that the Maillard reaction in C-AO was in the middle stage with browning substance production. Furthermore, the Maillard reaction was more advanced in C-Glu than in C-AO; however, it did not reach the advanced stage that causes the fragmentation of the reaction products [38].

In this study, the effect of glycation on collagen digestibility was monitored using gel permeation chromatography (Figure 2B). With the increase in reaction time, the molecular mass distribution of the digested collagens was changed in different ways. It was observed that the molecular mass of C-Sor was unchanged, indicating that the incubation step did not affect the digestibility of collagen. Furthermore, C-Glu was slightly enhanced in the high molecular mass part over the incubation time, suggesting that the proceeding Maillard-type glycation affected the collagen’s digestibility [39]. Moreover, unlike the tendency of C-Sor and C-Glu, it was observed that high molecular mass peptides increased in C-AO with progressing AO-glycation. This increase is proposed to have been caused by AO-glycation inhibiting collagen proteolysis and binding of the linear oligosaccharide AO to the peptide (mean molecular mass at mean DP = 6, which was approximately 1.1 kDa).

### 3.3. Antioxidant Activity of Glycated Collagen

DPPH and ABTS antioxidant assays are rapid and simple methods for evaluating the antioxidant activity of non-enzymatic antioxidants [40]. Therefore, to investigate the enhancing effect of glycation on the antioxidant activity of collagen digested in the gastrointestinal model, these digested collagens were subjected to DPPH and ABTS antioxidant assays.

**Figure 3:**
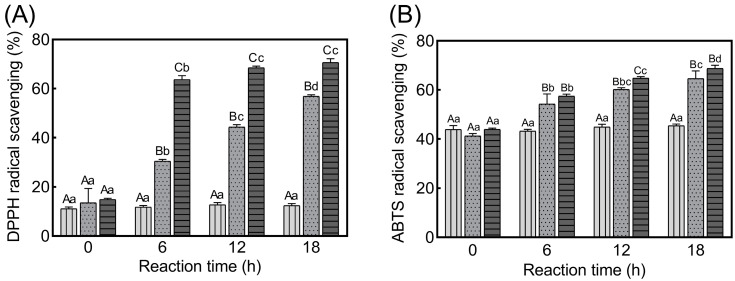
DPPH and ABTS radical scavenging activities of glycated collagens. (**A**): DPPH radical scavenging activity; (**B**): ABTS radical scavenging activity. C-AO (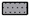), C-Glu (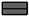), C-Sor (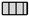). Different capital letters A–C indicate significant differences among the three collagen groups at the same reaction time. Different lowercase letters a–d indicate significant differences among the different reaction times in the same collagen group.

As shown in Figure 3A, the DPPH radical scavenging activity of C-Sor was about 12% and stayed at the same level during the incubation process, showing that the heat treatment did not affect the antioxidant activity of digested collagen. Alternatively, the DPPH radical scavenging activity was enhanced with glycation in the digested collagens, gradually raised in C-AO, and showed a 4.6-fold increase after 18-h glycation. Moreover, although the DPPH radical scavenging activity of C-Glu was enhanced quickly at the beginning of the glycation and reached 63% during the 6-h reaction, further glycation induced only a slight increase and finally reached approximately 70% after 18-h glycation.

Nevertheless, as presented in Figure 3B, the glycation effect on ABTS radical scavenging activity showed similar trends to DPPH radical scavenging activity; no change was observed in C-Sor. However, C-AO and C-Glu exhibited increasing ABTS radical scavenging activity with glycation progress.

Since AO and glucose alone had no marked radical scavenging activity (Figure S1), the enhancement of the antioxidant activity shown in Figure 3 was attributed to glycated collagen but not the conjugated sugars alone. Additionally, the reason for a more rapid improvement of its antioxidant activity for C-Glu than for C-AO is also proposed to be caused by the difference in reactivity between the two reducing sugars (Figure 1).

### 3.4. Comparison of Available Lysine Loss of Antioxidative Collagen between AO- and Glucose-Glycation

**Figure 4:**
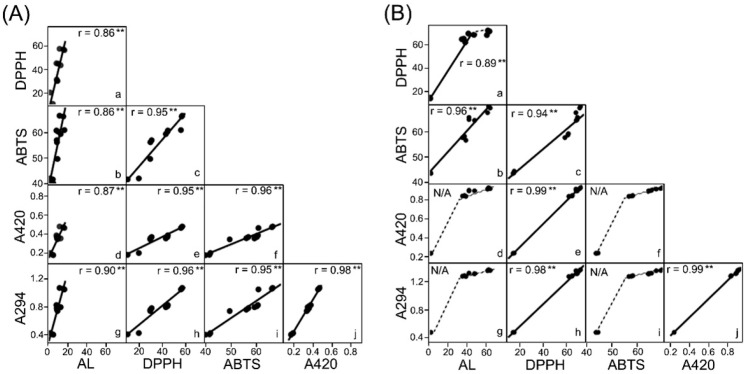
Relationship between antioxidant activities and Maillard reaction indicators in glycated collagens. Each box column presenting a bivariate scatterplot indicates mutual relationships between DPPH radical scavenging activity (DPPH), ABTS radical scavenging activity (ABTS), browning intensity (A420), the intermediate-stage indicator (A294), and available lysine loss (AL). Figures (**A**,**B**) present C-AO and C-Glu results, respectively. The numbers in the scatterplot also show the correlation coefficients (**: *p* < 0.01).

Columns a (DPPH vs. AL) and b (ABTS vs. AL) in Figure 4A,B showed a positive linear correlation between enhanced radical scavenging activity and available lysine loss (AL) in both glycated collagens, except for the lysine loss of more than 45% in DPPH-AL (Column a in Figure 4B). This exception (AL > 45%) proposes that the acquisition of DPPH radical scavenging activity in the C-Glu group was terminated. Furthermore, the slope of the regression line in C-AO (DPPH vs. AL: 3.35, ABTS vs. AL: 1.78) was about 3–4 times higher than that in C-Glu (DPPH vs. AL: 1.3, ABTS vs. AL: 0.41). Therefore, these trends indicate that AO-based glycation is an effective molecular modification that imparts antioxidant activities to collagen more efficiently than Glu-based glycation without significant lysine loss.

The results of Figure 4A also showed C-AO data from columns (e) and (h), then columns (f) and (i); as observed, positive linear correlations existed between absorbance increase and enhanced radical scavenging activities. Therefore, this result indicates that the products in the early to middle stages of the Maillard reaction contributed to the acquisition of antioxidant activities in C-AO, where glycation progressed slowly (discussed in Section 3.3).

Additionally, as shown in Figure 4B, the DPPH radical scavenging activity of C-Glu was enhanced with increased A294 and A420 (columns (h) and (e)), which was the same as in C-AO. Whereas the ABTS radical scavenging activity continued to potentiate, and this rise continues, although the increase in A294 (i) and A420 (f) slowed down with further progress in the Maillard reaction. Many studies have reported that the ultraviolet absorption components diminish, and the browning reaction slows down when the Maillard reaction progresses to the late stage [5,28,29]. Therefore, the enhancement of antioxidant activity in C-Glu was proposed to involve the late-stage products of the Maillard reaction. These results indicate that different Maillard reaction products enhance the radical scavenging activity of DPPH and ABTS each. Furthermore, it is apparent that AO-glycation can provide antioxidant activities to collagen without proceeding to the late Maillard reaction stage. Therefore, when analyzing protein function during glycation using the Maillard reaction, it is necessary to discuss the involvement of both the number of bound sugar units and the degree of progression of the Maillard reaction.

Lysine is one of the essential amino acids for humans, and lysine loss poses nutritional problems in the application of the Maillard reaction during food processing [41]. It has also been reported that advanced glycation end-products can be negative health factors in biological systems [42,43]. However, as previously described, the AO-modified collagen obtained enhanced antioxidant activity with low AL under the early and moderate reaction stages, indicating that AO is an optimal reducing sugar to modify collagen considering its health benefits.

### 3.5. Cytoprotective Activity of Glycated Collagens on H_2_O_2_-Induced Cell Oxidative Damage

H_2_O_2_ exposure has been widely employed in cell oxidative stress assays [35] since H_2_O_2_ directly penetrates the cell membrane, injures biomolecules (DNA, lipids, and protein), and causes organelle and cell damage [22]. Similarly, several studies have performed the cell oxidative damage assay using macrophages, which are the main targets of pro-oxidant action [22,44]. Furthermore, the cytoprotective effect of collagen and its peptides has been reported [22,45]. However, the cytoprotective effect of collagen-based Maillard reaction products on cell oxidative damage is less studied. Thus, the 18-h glycated collagens, which exhibited the highest antioxidant activity in DPPH and ABTS radical scavenging assays (Figure 3), were subjected to cytoprotective assessments against cell oxidative damage.

**Figure 5:**
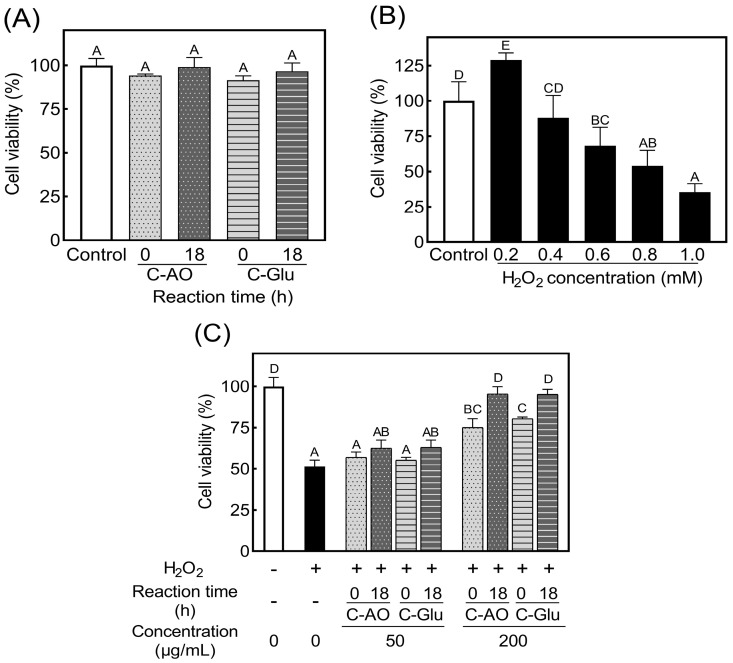
Cytoprotective effects of C−AO and C−Glu on oxidative damage in RAW 264.7 cells. (**A**): Cytotoxicity of glycated collagen. Pepsin–trypsin digested C−AO and C−Glu with glycation for 0 h and 18 h were added to RAW 264.7 cells, respectively. (**B**): Oxidative damage was induced by 0.2–1.0 mM H_2_O_2_ in RAW 264.7 cells. (**C**): The C−AO and C−Glu against cell oxidative damage; 50 or 200 µg/mL C−AO or C−Glu (0-h or 18-h glycation) were added to RAW 264.7 cells and stimulated by 0.8 mM H_2_O_2_. Data from additive-free cells were used as a control in calculating the cell viability of each experimental group. Bars with different letters indicate significant differences.

Before evaluating the cytoprotective effects of these glycated collagens, the cytotoxicity of glycated collagens was assessed using RAW 264.7 cells by adding the digested C-AO and C-Glu prepared by glycation for 0 h and 18 h. As shown in Figure 5A, the cell viability was unaffected in the presence of digested collagens, regardless of glycation. Next, the effect of H_2_O_2_ on cell viability was investigated to determine the appropriate H_2_O_2_ concentration. As shown in Figure 5B, cell viability after cultivation for 4 h diminished with increased H_2_O_2_ concentration, except for the 0.2 mM. Since the cell viability after adding 0.8 mM H_2_O_2_ decreased to 54% of the control value, which reached the median lethal dose (LD50), this condition was selected to conduct the H_2_O_2_-induced cell oxidative damage model [35].

Based on the preliminary experiment described above, the cytoprotective effect of the glycated collagens was demonstrated on the RAW 264.7 cells by treating the cells with 50 or 200 μg/mL of digested C-AO or C-Glu for 24 h before stimulation by 0.8 mM H_2_O_2_. The loss of available lysine in the 18-h glycated C-AO and C-Glu was 14.4% and 63.9%, respectively. As shown in Figure 5C, although no significant cytoprotective effect was observed with 50 μg/mL of the glycated collagens, the 18-h glycated collagens at 50 μg/mL showed higher cytoprotective activities than the 0-h glycated collagens (that is no reaction with collagen). Furthermore, when the concentration of C-AO and C-Glu was raised to 200 μg/mL, the cells treated with 18-h glycated collagens maintained high cell viability without loss induced by H_2_O_2_. The results in Figure 5C indicated that the glycation using the Maillard reaction effectively improves the cytoprotective effect on cell oxidative damage of collagen. AO-glycation also showed the possibility of obtaining similar health effects with a lower lysine loss than glucose-glycation.

### 3.6. Effect of Glycated Collagens on MDA, SOD, and CAT Levels in H_2_O_2_-Damaged Cells

To clarify the effects of C-AO and C-Glu on intracellular antioxidant enzymes and lipid peroxidation, the MDA, SOD, and CAT levels in RAW 264.7 cells with H_2_O_2_-induced oxidative damage were examined following the collagen treatments described in Figure 5.

**Figure 6:**
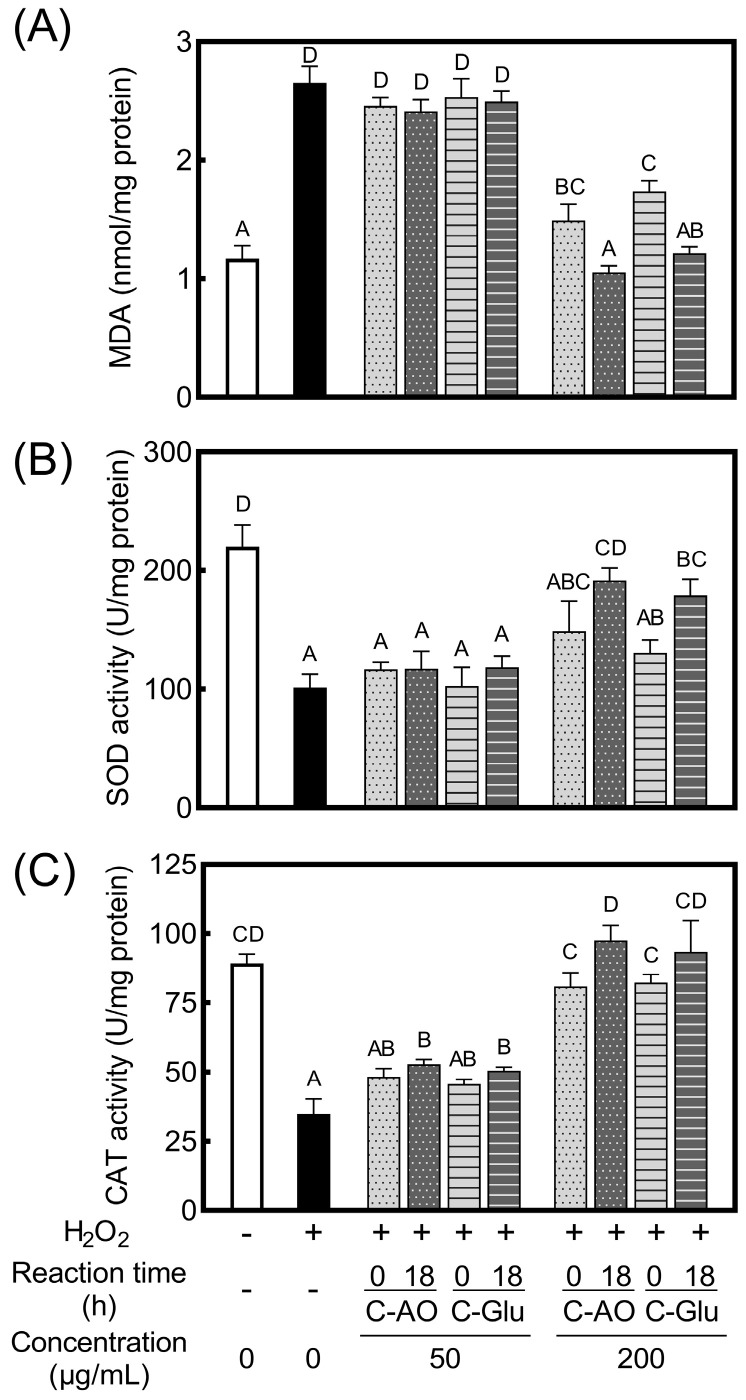
Effects of C−AO and C−Glu on the malondialdehyde (MDA), superoxide dismutase (SOD), and catalase (CAT) levels in RAW 264.7 cells with oxidative damage. (**A**): MDA content in RAW 264.7 cells with oxidative damage. (**B**): SOD activity in RAW 264.7 cells with oxidative damage. (**C**): CAT activity in RAW 264.7 cells with oxidative damage. All results were normalized to the protein concentration of the cell lysate (*n* = 3), and bars with different letters indicate significant differences.

MDA is a major product of lipid peroxidation, and an increase in the MDA levels suggests that cells have been exposed to oxidative stress [22,45]. As presented in Figure 6A, a marked increase in MDA content was observed in cells stimulated with only H_2_O_2_, indicating that the cells were exposed to oxidative stress, as discussed in Section 3.5. The MDA levels in H_2_O_2_-stimulated cells were not affected by treatment with 0-h or 18-h glycated collagens (C-AO and C-Glu) at 50 μg/mL, whereas the MDA levels were significantly reduced by treatment with 200 μg/mL 0-h glycated collagens. In addition, 18-h glycated C-AO and C-Glu at 200 μg/mL further reduced the MDA levels to the level without H_2_O_2_ stimulation. These results indicate that the cytoprotective activity of collagen was enhanced by AO- and glucose-glycation.

SOD and CAT are crucial endogenous antioxidant enzymes in cells, considered the first line of defense against oxidation, and play an important role in maintaining redox homeostasis in cells. SOD is the most powerful antioxidant enzyme in cells, and it catalyzes the superoxide anion to H_2_O_2_ [46]. CAT is a high-performance antioxidant enzyme that converts H_2_O_2_ into water and molecular oxygen, and it is a vital protective enzyme against oxidative stress-induced cell damage [46,47]. It has been reported that H_2_O_2_ stimulation reduced SOD and CAT activities in cells [22,45]. This phenomenon is also presented in Figure 6B,C, and treatment with 0-h or 18-h glycated collagens (C-AO and C-Glu) at 50 μg/mL imparted no protective effect to the loss of enzymatic activities due to H_2_O_2_ stimulation. However, the loss of SOD and CAT activities was markedly suppressed by 0-h glycated collagens at 200 μg/mL. Furthermore, the protective effect of 0-h glycated collagens on SOD and CAT activities was improved by treatment with 18-h glycated collagens. Consequently, their enzymatic activities were maintained at similar levels as those of unstimulated cells. The results in Figure 6 indicate that the cytoprotective effect of collagen was enhanced through Maillard-type glycation with AO or glucose, which was achieved by protecting the activity of endogenous antioxidant enzymes and reducing lipid peroxidation in a concentration-dependent manner. It has been reported that the cytoprotective activity of collagen against oxidative damage in cells was attributable to the protection of intracellular SOD and CAT activities and the suppression of lipid peroxidation [22,48]. The results of this study revealed that AO and glucose glycation improved these key actions in the cytoprotective effect of collagen.

## 4. Conclusions

Glycation with AO and glucose through the Maillard reaction enhanced the antioxidant activity and cytoprotective effect on the H_2_O_2_-induced cell oxidative damage of collagen; AO-glycation showed an effective improvement in antioxidant activity without a marked loss of available lysine. This study suggests that AO is an optimal reducing sugar for antioxidant production. Additionally, the findings of this study could contribute to the understanding of the enhanced antioxidant activity of collagen via Maillard-type glycation, further extending the application of Maillard-type glycation in collagen modification.

## Data Availability

The datasets generated for this study are available on request to the corresponding author.

## Supplementary Materials

The following supporting information can be downloaded at: https://www.mdpi.com/article/10.3390/foods11152374/s1, Figure S1: Reducing sugars have no marked effect on ABTS and DPPH radical scavenging activities.
