# Supplementary material for: Comparison of Maillard-Type Glycated Collagen with Alginate Oligosaccharide and Glucose: Its Characterization, Antioxidant Activity, and Cytoprotective Activity on H2O2-Induced Cell Oxidative Damage
Source: Foods. 2022 Aug 8;11(15):2374. doi: 10.3390/foods11152374 (PMC9367735; doi:10.3390/foods11152374)
Supplement: Supplementary file 1 [file foods-11-02374-s001.zip › foods-1732818-supplementary.pdf]

Supplementary Data

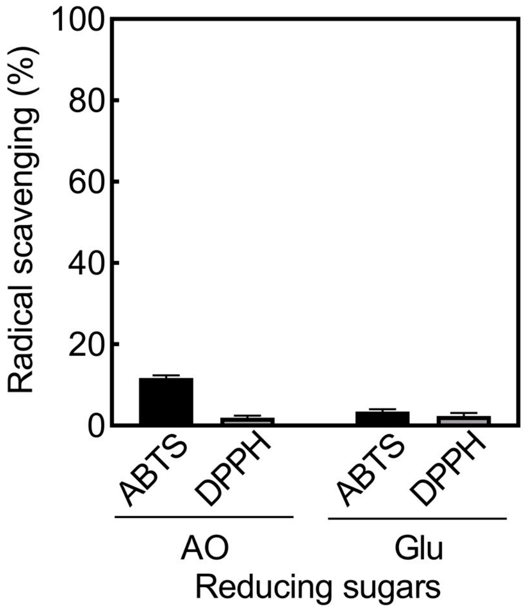

Figure S1. Weak or no effect of reducing sugars on ABTS and DPPH radical scavenging activities. 0.25 mg/mL and 5 mg/mL reducing sugars were employed in ABTS and DPPH assays, respectively. This concentration was the maximum sugar concentration, assuming that all reducing sugars (S) were bound to collagen (C) in the reaction mixture (C: S = 1: 0.5, w/w). Each assay was conducted independently three times.
